# Recent advances on biomass-fueled microbial fuel cell

**DOI:** 10.1186/s40643-021-00365-7

**Published:** 2021-02-09

**Authors:** Jamile Mohammadi Moradian, Zhen Fang, Yang-Chun Yong

**Affiliations:** Biofuels Institute, School of Environment and Safety Engineering, Jiangsu University, 301 Xuefu Road, Zhenjiang, 212013 China

**Keywords:** Biomass, Electricity generation, MFC, EAM, Sustainable energy

## Abstract

Biomass is one of the most abundant renewable energy resources on the earth, which is also considered as one of the most promising alternatives to traditional fuel energy. In recent years, microbial fuel cell (MFC) which can directly convert the chemical energy from organic compounds into electric energy has been developed. By using MFC, biomass energy could be directly harvested with the form of electricity, the most convenient, wide-spread, and clean energy. Therefore, MFC was considered as another promising way to harness the sustainable energies in biomass and added new dimension to the biomass energy industry. In this review, the pretreatment methods for biomass towards electricity harvesting with MFC, and the microorganisms utilized in biomass-fueled MFC were summarized. Further, strategies for improving the performance of biomass-fueled MFC as well as future perspectives were highlighted.

## Introduction

In the twenty-first century, there is an urgent need for renewable energy due to the rapid depletion of fossil fuels and the increasing concerns about pollutions. Thus, various countries are looking for alternative resources such as biomass as a reliable, sustainable, and a more benign resource to reduce the demands on fossil fuels. Biomass is one of the most abundant sources among the various types of new energy sources (Alidrisi and Demirbas 2016; Menandro et al. 2017). Moreover, the use of biomass energy could be considered as a carbon–neutral process as the CO_2_ emission is equal to or even lower than that biomass fixed from the atmosphere. On a global scale, biomass rank fourth as an energy resource, which could provide approximately 14% of the world’s energy needs (O’Mahoney et al. 2013). To date, biomass could be transformed into different kinds of energy products such as heat, gas, fuels, and electricity. Among them, electricity from biomass has been highly regarded in terms of its high capability to various aspects of life and industry (Moqsud et al. 2014). Among the various approaches for biomass conversion to electricity, microbial fuel cells (MFC) attracted much attention due to its high theoretical energy efficiency (non-Carnot limited) and mild operation conditions requirement (Bullen et al. 2006; Mathuriya and Yakhmi 2016).

**Fig. 1:**
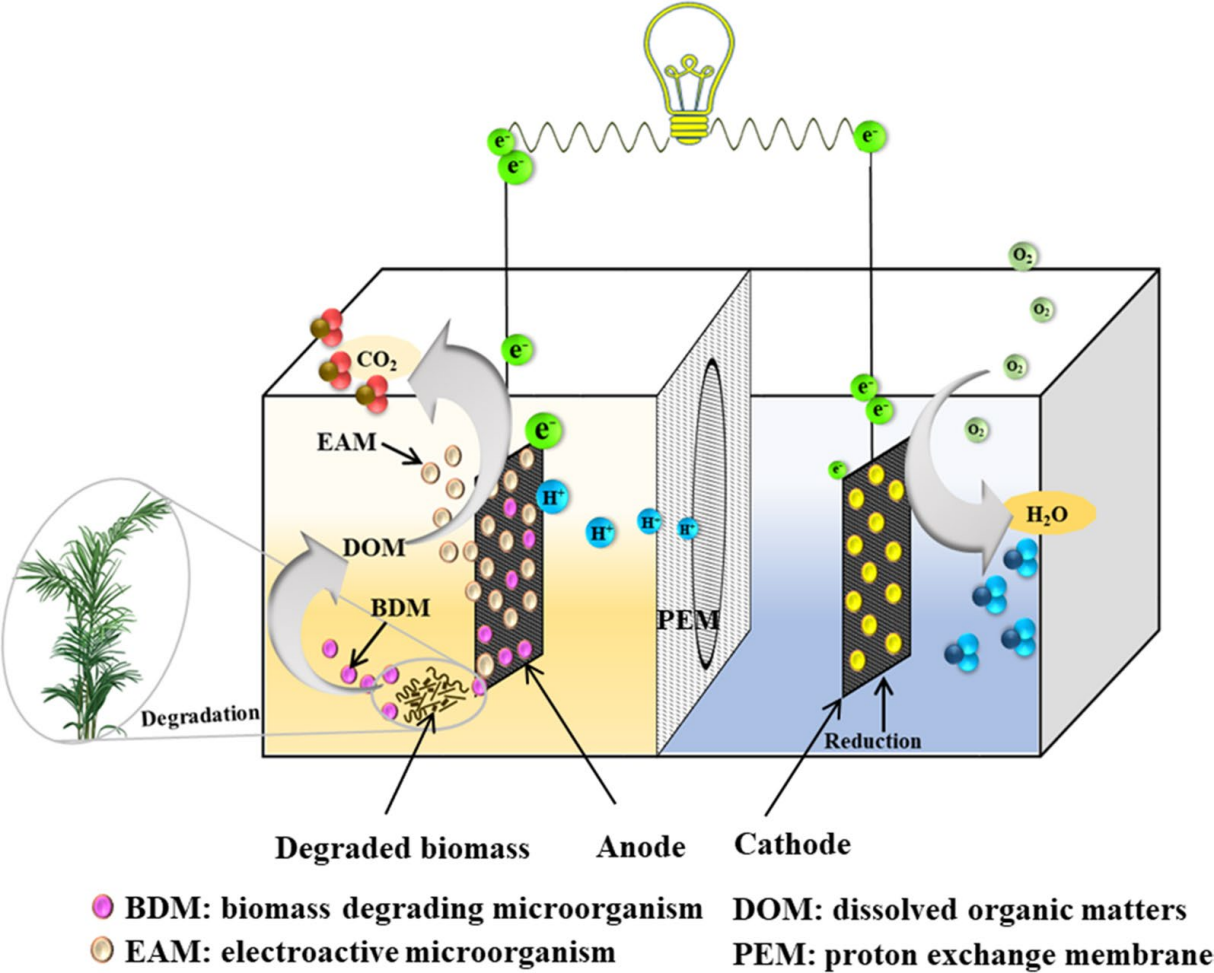
Model for biomass-fueled proton exchange membrane (PEM) separated microbial fuel cell (MFC). The electroactive microorganisms (EAMs) formed EAMs biofilm and were responsible for the degradation of biomass in the anode

MFC can efficiently harness the energy stored in the chemical bonds of organic compounds through catalytic reactions by microorganisms, which can directly convert the chemical energy into electrical energy (Yong et al. 2013; Yu et al. 2020). MFC relies on the unique microorganism called electroactive microorganisms (EAMs) that can degrade the organic matters with diverse metabolic pathways and pass the released electrons onto the anodic electrode. The collected electrons are then transferred to the cathode through the external electric circuit and electricity is generated (Logan et al. 2006; Thygesen et al. 2011) (Fig. 1). In this case, the organic matters were directly converted into electricity with the MFCs. As this biological energy conversion process avoided the Carnot cycle (which limited the energy efficiency of thermo-electricity conversion processes), it is expected to achieve high efficiency (Hassanzadeh and Mansouri 2005). More impressively, this energy conversion process with MFC is a biological process that can be conducted in mild conditions, which makes it promising for various applications. Although the power output of MFC is still low and should be substantially improved, it already showed attractive applications as a micro-power supplier for biosensors, a long-term power supplier in remote area, deep sea, or outer space (ElMekawy et al. 2018; Ivars et al. 2018).

As biomass is an energy-rich and abundant resource, it is expected to be an ideal fuel for MFC and MFC would also be an attractive energy conversion technology for the biomass industry. Therefore, many efforts have been made to develop and optimize biomass-fueled MFC during the past years. In general, as most of the microorganisms cannot directly digest biomass, pretreatment is usually required as an essential process for biomass-fueled MFC. Various EAMs for biomass-fueled MFC were isolated and characterized due to EAM is the key player in electricity generation. Besides, process optimization for performance improvement of MFC also attracted much attention. In this paper, various biomass substrates and EAMs used for biomass-fueled MFC were summarized, recent progress on biomass pretreatment and performance optimization for biomass-fueled MFC were also highlighted.

## Biomass substrates and EAMs used for biomass-fueled MFCs

### Biomass substrates used in MFCs

To date, various biomass including chitin (Li et al. 2017a, b), kitchen waste (Hou et al. 2016; Moqsud et al. 2014), orange peels (Miran et al. 2016a), algal biomass (Gajda et al. 2015; He et al. 2014), forest detritus in the forested wetland (Dai et al. 2015), wheat straw hydrolysate (Song et al. 2014; Thygesen et al. 2011), rice straw (Hassan et al. 2014), corn stove (Wang et al. 2017; Zhang et al. 2013), solid potato wastes (Du and Li 2017; Du et al. 2017), food waste (ElMekawy et al. 2015; Li et al. 2016), corn stalk biomass (Liu et al. 2015), lemon peel (Miran et al. 2016b), cow dung (Bharadwaj and Kumar 2012; Javalkar and Alam 2012) has been exploited as fuel sources for bioenergy production in MFCs (Table 1). The biomass used can be categorized as lignocellulosic biomass (cellulose, hemicellulose, and lignin, such as wood*,* sugarcane bagasse*,* rice husk*,* rice straw*,* corn cob, etc*.*) and non-lignocellulosic biomass with primary components composed of lipids*,* proteins*,* starch*,* inorganics, and minerals*.* Non-lignocellulosic biomass includes sewage sludge*,* animal manure, algae*,* etc. The quality of biomass is often determined by its inherent characteristics such as moisture content, energy yield, bulk density, size, and shape (Tursi 2019), which influence the bioconversion processes and energy production capacity in MFCs. According to these researches (Table 1), most of raw biomass or organic wastes derived biomass could be exploited as fuels for MFCs, which could imply that MFC would be extensively adaptable for energy harvesting from biomass.

**Table 1:** Performance of MFCs fueled with various biomass substrates

Biomass	Microorganism	Pretreatment method	Anode	Electrode modification	MFC performance	Refs.
Chitin	*Shewanella oneidensis-*MR-1	Physical pretreatment by the mechanical process of size reduction	Carbon felt	None	4.24 µA/cm^2^	Li et al. (2017a, b)
Cattle manure	Escherichia coli strain K-12 (ATCC25257), and manure leachate	Untreated	Brush carbon	None	93 mW/m^2^	Zheng and Nirmalakhandan (2010)
Raw rapeseed straw	Post-fermentation sludge	Hydrothermal pretreatment, microwave treatment, and enzymatic hydrolysis	Graphite felt	The cathode (graphite felt) additionally coated with a Pt/C catalyst	54 mW/m^2^	Jablonska et al. (2016)
Orange peel waste	Anaerobic wastewater sludge	Physical pretreatment by the mechanical process of size reduction	Graphite felt	None	358.8 ± 15.6 mW/m^2^	Miran et al. (2016a)
*Enteromorpha prolifera* bloom	Pre-domesticated bacteria	Acid hydrolysis	Carbon cloth	None	1027 mW/m^2^	Wang et al. (2013)
Rice straw	Mixed-culture of cellulose-degrading bacteria (CDBs)	Physical pretreatment by the mechanical process of size reduction	Carbon paper	None	190 ± 13.6 mW/m^2^ 146 mW/m^2^	Gurung and Oh (2015), Hassan et al. (2014)
Kitchen waste anaerobically digested effluent (KWADE)	Five species of microalgae including *Golenkinia* sp. SDEC-16, *Scenedesmus* SDEC-8, *Scenedesmus* SDEC-13, *S. capricormutum,* and *Chlorella vulgaris*	Physical pretreatment by the mechanical process of filtration to remove insoluble solids	Graphite	None	6255 mW/m^3^ inoculated with *Golenkinia* SDEC-16	Hou et al. (2016)
Food wastes	Anaerobic sludge	Physical pretreatment by the mechanical process of size reduction	Carbon cloth	Carbon cloth-based cathode containing 10% platinum	5.6 W/m^3^	Li et al. (2016)
Corn Stover waste	Domestic wastewater	Acid hydrolysate, and steam-explosion	Carbon paper	None	93,971 mW/m^2^ (acid hydrolysates)	Zuo et al. (2006)
Solid potato waste	Activated sludge	Physical pretreatment by the mechanical process of size reduction	Carbon felt	None	6.8 mW/m^2^	Du and Li (2017)
Corn straw	*Shewanella onidensis* MR-1	Physical pretreatment by a mechanical process of size reduction, and acid hydrolysate	Carbon cloth	Polyaniline (PANI) nanowire	660 mW/m^2^	Wang et al. (2017)
Corncob pellets	Ruminal contents, Pasteurized soil, and *Geobacter metallireducens*	Untreated	Graphite brush	None	230 mW/m^3^	Gregoire and Becker (2012)
Macroalgal biomass (*Laminaria digitate*)	Domestic wastewater	Physical pretreatment by a mechanical process of size reduction (< 2 mm), and grounded into powder	Carbon brush	None	0.5 V	Zhao et al. (2018a, b)
Lipid extracted algal	Pretreated cow manure	Methanol-chloroform (2:1) by modified Bligh and Dyer extraction method	Graphite felt	None	2.7 W/m^3^	Khandelwal et al. (2018)
Cornstalk	Municipal wastewater	Hydrothermal liquefaction (HTL)	Carbon felt	CNTs	680 mW/m^3^	Liu et al. (2015)
*Canna indica* (canna)	Rumen microorganisms	Untreated	Carbon paper	None	0.405 W/m^3^	Zang et al. (2010)
Sugarcane bagasse, Corn cob	Two acetic acid bacteria, namely *A. aceti* (NCIM no. 2116) and *G. roseus* (NCIM no. 2049)	Biological pretreatment by *O. annae*, a freshwater cyanobacterium	Carbon felt	None	8.78 W/m^3^ 6.73 W/m^3^	Krishnaraj et al. (2015)

### Various EAMs used in MFCs

EAMs based on their microbial community can differ as pure-culture, and mixed-culture. Pure-culture EAMs have been characterized with a diversity of microorganisms in MFCs belonging to three domain phyla including bacteria in the Firmicutes such as *Clostridium butyricum* and Actinobacteria such as *Actinoalloteichus cyanogriseus*, and in all classes of Proteobacteria such as *Geobacter sulfurreducens*, archaea such as the hyperthermophile *Pyrococcus furiosus* and eukaryotes such as *Cystobasidium slooffiae* JSUX1. Although pure-culture EAMs is useful to clarify the EET mechanism in MFCs, it requires relatively strict operating conditions and can only utilize specific compounds in hydrolysates (Cao et al. 2019). To date, various pure-culture EAMs including Gram-positive bacteria (Wrighton et al. 2011), Gram-negative bacteria (Hasan et al. 2017; Li et al. 2017a; Wang et al. 2017), yeast (Moradian et al. 2020; Sayed and Abdelkareem 2017), and even fungi (Sekrecka and Toczyłowska 2018) have been reported in the literature (Table 1). The existence of at least one EAM is required to produce energy in MFCs. However, a diversity of EAMs can contribute to more efficient energy production. In most cases, inoculated MFCs with mixed-culture such as activated sludge due to its high microbial diversity often generate much greater PD and utilize biomass hydrolysate more efficiently. Vajda et al. (2014) operated xylose (main sugar from biomass) fueled MFC using *Shewanella putrefaciens* and mesophilic anaerobic sludge as inoculums. Despite both pure-culture and mixed-culture MFCs were able to metabolize and generate electricity from xylose, but MFC with mixed-culture could perform higher PD. So far, investigation implied that hydrolyzed biomass can be utilized by pure-culture EAMs in MFCs, while for raw biomass without pretreatment, the inoculum for MFCs usually should be derived from mixed-culture that contained CDBs (responsible for cellulose degradation) and EAMs (responsible for electricity generation). Also, cellulose-degrading bacteria (CDBs) consortia can be used as a biocatalyst to partially degrade the lignocellulosic biomass and produce electricity. In some cases, industrial or municipal wastewater contained CDBs was also used as an active inoculum for biomass-fueled MFCs. For example, Hassan et al. (2014) developed a dual-chambered MFC fueled with rice straw without pretreatment using CDBs as biocatalyst. With an initial rice straw concentration of 1 g/L, the PD reached 145 mW/m^2^ (Hassan et al. 2014).

## Biomass pretreatment for MFCs

**Fig. 2:**
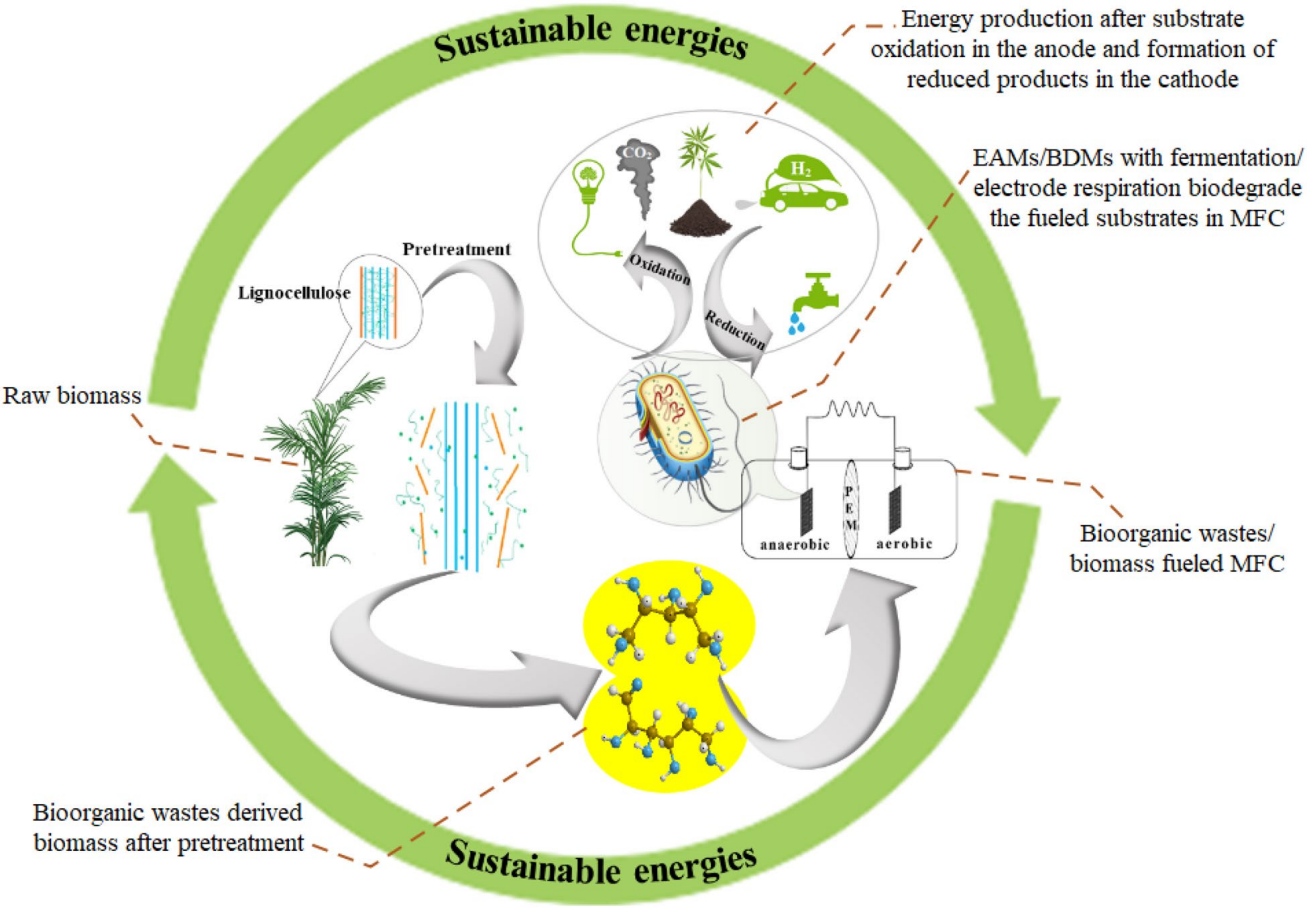
Overview of processes for energy production from biomass in MFC. Pretreatment technique could break down the lignocelluloses to reducing sugars, which served the fuels for energy production by EAMs

Most microorganisms are not capable of efficient hydrolysis of biomasses such as lignocellulosic biomass in the anode chamber, which results in a low PD for MFCs (Pant et al. 2010). The recalcitrance of lignocelluloses to fermentable sugars is the major technical hindrance. Especially, the hierarchical structure of biomass can resist chemical or enzymatic attack for releasing of fermentable sugars (Wegner and Jones 2009). Crystalline cellulose is highly ordered due to the existence of strong hydrogen bonding structure, which is very stable and difficult to be permeated (Klemm et al. 2005). To overcome these limitations, pretreatment processes must be used to release various reducing sugars (Fig. 2). However, the efficiency of the pretreatment is depending on the characterization of biomass, which can be considered as a factor to maximize product recovery for the biorefinery process in MFCs.

*Physical pretreatment* techniques operated with mechanical processes including chipping, milling, and grinding can reduce particle size, break down the crystallinity and degree of polymerization, which substantially improve the biodegradability of biomass in MFCs. It has been affirmed that the use of a fermentation media contained solid substrate gave a low PD due to slow hydrolysis of the biodegradable materials, which indicated that particle size is an essential factor for maximum derived bioenergy production. Also, it has been reported that further reduction of biomass particle size below 40 mesh has some effects on the hydroysis rates and yields, which results in a large quantity of the available material in the biodegradation process in MFCs (Agbor et al. 2011). Moreover, different types of irradiation processes (e.g., ultrasonication, electron beams, X-rays, or gamma rays) can be used for the physical pretreatment of biomass. Shen et al. (2018) reported the effect of ultrasonic pretreatment on electricity generation with dairy manure (DMMFC) as the main substrate in the DMMFC. The pretreated DMMFC obtained a maximum PD of 102 mW/m^2^ at the ultrasonic power of 600 W, which was 241% higher than the untreated substrate (Shen et al. 2018). Tao et al. (2013) confirmed ultrasonication could be an efficient pretreatment strategy for vegetable or grass wastes. The pretreated vegetable wastes with ultrasonication at > 1.0 W/mL could attain a maximum PD and COD removal of 10.19 W/m^3^ and 62.5%, respectively, while these values with the untreated sample were only 5.96 W/m^3^ and 35.1%, respectively (Tao et al. 2013).

*Acidic pretreatment* is one of the most commonly used methods among various chemical pretreatment techniques. Pretreatment with acid hydrolysis can improve the enzymatic hydrolysis efficiency and enhance the energy conversion efficiency of lignocellulosic biomass in MFCs. To date, these acids including concentrated mineral acid (CA), dilute mineral acid (DA) and dicarboxylic acid have been used for lignocellulosic biomass pretreatment. Among them, CA such as H_2_SO_4_ and HCl are potent agents for lignocellulosic biomass. However, these acids are toxic, corrosive, and hazardous which requires specific  reactors that can resist corrosion. Meanwhile, the dilute sulfuric acid pretreatment showed high reaction rate for lignocellulose hydrolysis. Initially, two types of dilute acid hydrolysis pretreatment methods have been developed: high temperature (*T* > 160 °C) and low temperature (*T* < 160 °C) (Sun and Cheng 2002). Although a high temperature in the DA hydrolysis is desirable for cellulose hydrolysis, the saccharification yield is low due to sugar decomposition (McMillan 1994). Wang et al. (2017) used the diluted sulfuric acid pretreated corn straw as fuel for direct electricity generation in MFC inoculated with pure-culture. The maximum PD delivered by this MFC was 17.2 ± 0.3 mW/m^2^, which showed the feasibility of biomass hydrolysate as the potential of electrical production using pure-culture in MFC. The combination of electrode modification and electron shuttle addition could also result in a high PD of 660 mW/m^2^ from the hydrolysate with a pure-culture of *Shewanella oneidensis* MR-1 (Wang et al. 2017). In another study, Wang et al. (2013) pretreated *Enteromorpha prolifera* biomass with 2% sulfuric acid at 100 °C for 4 h. The acid hydrolysate of *E. prolifera* biomass contained a total of 8.5 g/L monosaccharides and had the high content of l-rhamnose (3.74 g/L), followed by d-xylose (2.11 g/L), d-glucose (1.78 g/L), d-glucuronic acid (0.581 g/L), and d-glucuronic acid lactone (0.29 g/L). The PD of 1027 mW/m^2^ at a current density of 3.8 A/m^2^ was achieved with an initial hydrolysate concentration of 1000 mg COD/L in MFC. Also, the Coulombic efficiency (CE) and COD removal of 69.1% and 76.1% were obtained, respectively (Wang et al. 2013). Zhang et al. demonstrated that stable PD could be generated from wheat straw hydrolysate, in which the PD reached 123 mW/m^2^ with an initial hydrolysate concentration of 1000 mg COD/L (Zhang et al. 2009).

*Alkali pretreatment* involves the use of base reagents such as sodium hydroxide (NaOH), hydrazine, anhydrous ammonia, potassium hydroxide (KOH), or lime (Ca(OH)_2_). Although this method can be operated at ambient temperature, the reaction time is usually long, e.g., from hours to days (Amin et al. 2017). Alkali pretreatment is preferable for low lignin content biomass such as agricultural residues/grasses, and higher lignin content substrates such as woody biomass are unsuitable for this method. Song et al. (2018) demonstrated that sodium hydroxide (NaOH) could be used for rice straw pretreatment for a solid phase microbial fuel cell (SMFC). The SMFC with pretreated-rice straw using NaOH (5%) could retain a maximum PD of 140 mW/m^2^, which was 3.6 times higher than the untreated SMFC (Song et al. 2018). Xiao et al. also confirmed the feasibility of alkaline pretreatment for sludge-fueled MFC, which was obtained a PD of 46.82–55.88 mW/m^2^ with a fast alkaline treatment at a high concentration of sodium hydroxide (Xiao et al. 2013).

*Biological/enzymatic pretreatment* is a remarkable achievement that advocates a limited or no generation of toxic substances, eco-friendly process with low energy requirement, and mild operating conditions. Some bacteria and fungi producing cellulases can hydrolyze and disrupt the crystalline structure of lignocellulosic biomass and increase sugar yields to improve the performance of MFCs (Wagner et al. 2018). Bacterial species belonging to *Clostridium*, *Cellulomonas*, *Bacillus*, *Thermomonospora*, *Ruminococcus*, *Bacteriodes*, *Erwinia*, *Acetovibrio*, *Microbispora*, and *Streptomyces* can produce cellulases (Sun and Cheng 2002). White- and soft-rot fungi have been demonstrated to degrade lignocellulosic biomass. White-rot being the most effective method for biological pretreatment of biomass (Agbor et al. 2011; Sun and Cheng 2002). Brown-rot fungi can mainly attack cellulose, whereas white and soft-rot attack both lignin and cellulose via the production of enzymes (for example, lignin peroxidases, polyphenol oxidases, manganese-dependent peroxidases, and laccases)(Agbor et al. 2011). In addition to the three major types of cellulase enzymes, there are also a number of auxiliary enzymes that can attack hemicelluloses, such as glucuronidase, acetylesterase, xylanase, β-xylosidase, galactomannanase, and glucomannanase (Sun and Cheng 2002).

Nevertheless, the rate of biological hydrolysis is usually too slow. The disadvantages of this method includes the requirement of a long retention time ranging from 10 to 14 days, a careful control of growth condition avoiding contamination, and a substantial amount of space for conducting biological pretreatment are considered this method less applicable commercially. Krishnaraj et al. (2015) applied a new strategy of a novel three-chamber MFCs for simultaneous degradation of lignocellulosic biomass (sugarcane bagasse and corncob) and bioelectricity generation. The lignocellulose was first degraded by *Oscillatoria annae* from a freshwater cyanobacterium in the first compartment of the three-chamber MFCs. Co-culture of *Oscillatoria annae* and *Gluconobacter roseus* were used as the anodic inoculums for electricity generation using the decomposed substrates from the first chamber in MFCs. The maximum PD was 8.78 W/m^3^ and 6.73 W/m^3^ with sugarcane bagasse and corncob as substrates, respectively (Krishnaraj et al. 2015). Also, enzymatic hydrolysis has been extensively applied for biological pretreatment. In a study, Rezaei et al. (2008) used cellulases enzyme for enzymatic hydrolysis of cellulose coupled with electricity generation in MFCs. The maximum PD with cellulases enzyme reached 100 mW/m^2^.

## Performance improvement for biomass-fueled MFCs

### Operation conditions and optimization

The specific metabolic process in MFCs varies on the type of biomass/organic wastes and the operational conditions of MFCs (Guo et al. 2020). In this section, operation conditions (pH, temperature, and organic loading rate), and optimizations for performance improvement in MFCs are discussed in the following.

*pH* is one of the essential factors in MFCs that affects both anodic microbial activities and cathodic reactions. Accumulation of protons would cause anolyte acidification, and electrolyte alkalization in the cathode chamber. Hence, reducing pH in the anode chamber due to increased proton concentration results in low power production, which represses the EAMs activation. On the contrary, it causes an increased pH in the cathode chamber that inhibits the oxygen reduction reaction (ORR) (Ivars et al. 2018). Although the use of pH buffers such as phosphate or bicarbonate (pH 7.0) has been suggested for controlling pH at the anolyte, it may increase operating costs and effluent desalination or further phosphorous removal burden (Chen et al. 2019). It was found that an increased anodic pH shall attribute to an increased COD removal and improve the performance of MFCs. The optimal pH strongly depends on the type of microorganisms. Zhang et al. (2011) studied the effect of pH on the performance of MFC and anodic microbial community. Results showed a faster COD removal under acidic pH conditions, in which *Simplicispira*, *Variovorax*, *Comamonas*, and *Acinetobacter* were the major communities under acidic conditions. Anodic biofilm cracked and cell number greatly decreased at pH ≤ 5.0, and further, MFCs was failed at pH 4.0 due to microbial community composition changes. However, the MFCs could recover optimal electricity generation when pH was further readjusted to 7.0 (Zhang et al. 2011). Optimal pH for maximum power production reported was 8–10 in an air-cathode MFC fueled with acetate (Zhao et al. 2017). Usually, the anodic microbial reaction preferred a neutral pH for optimum cell growth, whereas a weak alkaline pH was more appropriate for cathodic reaction.

*Temperature* effect depends on the nature of anodic EAMs and the characteristics of biomass in MFCs. It has been reported that microorganisms can grow in four classified optimal growth temperature, i.e., psychrophiles (10–15 °C < 20 °C), mesophiles (25–40 °C < 45 °C), thermophiles (50–85 °C), hyperthermophiles (80–113 °C) (Stetter 2006). However, most of the characterized EAMs belong to mesophilic classification. At extremely low temperatures, microbial reactions slow down, and eventually, MFCs cannot be operated in most cases (Ivars et al. 2018). However, MFCs with psychrophiles EAMs can operate at low temperature and attain high CE. Behera et al. (2011) evaluated temperature effects on the performance of dual-chambered mediator-less MFC by adjusting the temperature between 20 and 55 °C. The highest COD removal efficiency of 84% was observed at an operating temperature of 40 °C. Tee et al. (2018) studied the performance of MFC with an adsorption system (MFC-AHS) and palm oil mill effluent as a substrate under various operating temperatures. The optimum operating temperature for such a system was found at 35 °C. Also, results revealed that the maximum current density could increase linearly with the temperature at a rate of 0.1772 mA/m^2^/°C, whereas maximum PD was in a polynomial function (Tee et al. 2018). Larrosa et al. (2010) investigated single-chambered and dual-chambered MFC operation at different temperatures ranging from 4 to 35 °C. The results revealed that the temperature as a crucial factor for COD removal and bioelectricity production, which were obtained 58% COD removal with maximum PD of 15.1 mW/m^3^ reactor (8.1 mW/m^2^ cathode) at 4 °C, and 94% COD removal with maximum PD of 174 mW/m^3^ reactor (92.8 mW/m^2^ cathode) at 35 °C (Larrosa-Guerrero et al. 2010).

*Organic loading rate (OLR)* has a significant impact on anodic biofilm, which primarily depends on the chemical characteristics of wastes. Especially, the fermentation of biomass/organic wastes can result in acidic metabolites production, which affects the anodic electrolyte. Therefore, the OLR fueled MFCs should be carefully optimized to achieve high performance. Further, it is confirmed that PD and CE in MFCs are closely related to OLR, in which an increased or decreased OLR can affect the efficiency of electron transfer. Operation of MFCs at the higher OLRs usually resulted in a decreased CE (Velvizhi and Mohan 2012). In a study reported for an MFC with treating leachate, the increasing OLR from 0.65 to 5.2 kg COD/m^3^/day resulted in a decrease of overall CE from 14.4 to 1.2% (Zhang et al. 2008). Cetinkaya et al. investigated the effect of OLR with changing HRT and leachate COD concentration. The results indicated the COD removal and current density were significantly affected by increasing OLR, although the performance of MFC decreased when HRT was reduced (Cetinkaya et al. 2016).

### Ionic conductivity of the electrolyte

Maintaining a suitable pH condition of electrolyte is necessary for obtaining a high PD and CE in MFCs. Cations such as Na^+^ and K^+^, other than H^+^ are prone to transfer toward the cathode, however, H^+^ mass transport is sluggish, which its accumulation in the anode causes anolyte acidification, and significantly restricts the electricity generation in the MFCs (Ren et al. 2017). Eliminating the anolyte acidification with alkaline catholyte through electrolyte recirculation has relieved the pH decline in MFCs, whilst O_2_ was likely to be influenced to the anode and restricted the activity of anode biofilm (Zhang et al. 2015). Further, inorganic ions buffers are always indispensable in MFCs to provide certain ionic conductivity and maintain stable pH conditions of the electrolyte (Chen et al. 2019). In addition, inorganic carbons (IC) such as H_2_CO_3_ (dissolved CO_2_), HCO_3_^−^, CO_3_^−2^ could be produced as the final metabolites of MFC, which are considered as endogenous buffers, although their accumulation concentration is insufficient to prevent acidification of anolyte (Ren et al. 2017). To overcome this limitation, Ren et al. (2017) reported a novel buffer-free MFC with anolyte recycling as a feasible strategy that could increase the IC concentration of the anolyte, thoroughly eliminating anolyte acidification and dramatically enhancing the electric power of MFCs.

### Electrode modification

Electrode materials should possess good electron conductivity, large surface area and good biocompability for microbial adherence. The surface properties of an electrode, such as roughness, porosity, and surface hydrophilicity can affect the formation of biofilm and subsequently derived electric power in MFCs (Zhao et al. 2017). In recent decades, carbonaceous is the most extensively used anodic material in MFCs. Carbonaceous-based materials such as carbon paper (Hassan et al. 2014), granular graphite (Habibul et al. 2016; Vilajeliu et al. 2017), and graphite rods (Xu et al. 2015) have been identified and widely used in biomass-fueled MFCs. However, the commercial carbon-based electrode showed a smooth surface with low electrochemical activity and biocompatibility. Hence, various strategies for electrode surface modification have been developed. For example, Chen et al. (2018) developed the candle soot modified-CC electrode by inoculating *Aeromonas hydrophila* NIU01 in MFCs. The modification with 60-s could alter the hydrophobic surfaces of the CC electrodes to super-hydrophilic. Further, the electrochemical measurement of the modified electrode exhibited the highest PD of 19.8 ± 0.2 mW/m^2^ with an internal resistance of 619 Ω, which was higher than that of MFCs conducted with the bare electrodes (10.2 ± 0.2 mW/m^2^) (Chen et al. 2018). In another study, Zhao et al. (2018a, b) thermally modified CF electrodes with a mixed solution of concentrated HNO_3_ and 30% H_2_O_2_ in different volume ratios. The inoculum of MFCs was supplied from local domestic sewage. The modification decreased the anodic charge transfer resistance with a maximum PD of 785.2 mW/m^2^, which was 51.1% higher than the bare electrodes in the MFCs (Zhao et al. 2018a, b). Moreover, modification with conductive polymers such as polypyrrole and polyaniline was demonstrated for improving anodic biofilm formation. The polymer composites can increase electrode surface roughness, and also the presence of cationic nitrogenous groups in their composite structure can enhance cellular adhesion electrostatically (Fogel and Limson 2016). For example, Li et al. (2018) introduced the polypyrrole nanowires coated by graphene oxide (PPy-NWs/GO) using a one-step electrochemical method. The performance of PPy-NWs/GO showed higher PD than PPy-NWs. Besides, the PPy-NWs/GO showed a more extensive biofilm of microbial attachment, which was owing to the GO nanosheet (Li et al. 2018). Razalli et al. (2017) pretreated the extracted crystalline nanocellulose of semantan bamboo with acid hydrolysis to synthesize a polyaniline/crystalline nanocellulose (PANI/CNC) electrode via in situ oxidative polymerization of aniline. The EIS results of PANI/CNC displayed a lower value of R_CT_ (148 Ωcm^2^) compared to the bare (177 Ωcm^2^) and PANI (156 Ωcm^2^) electrodes, which revealed that PANI/CNC incorporation could significantly reduce the charge transfer rate (Razalli et al. 2017). Moreover, nanoparticles, through a combination of the improved electrode surface, alteration of surface chemistry, and the presentation of electroactive moieties to the microbial cells have been used for improvements of the electrical current in MFCs (Fogel and Limson 2016). Ni, Pd, Au, and Fe_2_O_3_ nanoparticles have been used to enhance the direct EET for performance improvement of MFCs (Fogel and Limson 2016). Further, carbon-based nanomaterials including carbon nanotubes (CNTs), carbon nanoparticles, and graphene have also been performed for improving cell/electrode interaction, and enhancing EET pathway in MFCs. Besides, the rational inclusion of nanomaterials as electrode material/modifiers could significantly improve the electricity generation of biomass-fueled MFCs. Graphene oxide (GO) with rich hydrophilic functional groups and possessing biocompatibility, superior electrical, mechanical, and optical properties has developed a strong electrochemical performance in MFCs (Yong et al. 2014). GO can react with organic or inorganic chemicals and to remove the oxygen atoms to form proxy groups, which results in reduced graphene oxide (rGO) sheet network. However, apart from chemical reduction of GO, there are many techniques (i.e., hydrothermal reduction, electrochemical reduction, solvothermal reduction, and microbial reduction) that can react with GO and expose the conjugated *sp* network and degrade the electrical properties of the GO dispersion to form rGO nanosheet (Yong et al. 2014). Nevertheless, the toxicity of some agents cannot be neglected. For example, chemical reduction requires a potent reducing reagent such as hydrazine hydrate (N_2_H_4_), which is a highly corrosive material. Hence, the new strategies of GO reduction with biocompatible property and under mild-condition have been considered in recent studies. For example, Goto et al. (2015) reported the effect of GO on SMFCs (sediment) and PMFCs (plant) at different concentrations. Findings revealed a biological GO reduction after 10 days in GO-SMFCs under anaerobic incubation. The highest PD of GO-SMFCs containing 1.0 g/kg of GO was 40 ± 19 mW/m^2^. On the contrary, the GO reduction in PMFCs was much slower than GO-SMFCs, which exhibited a reduction in GO after 27 days of operation time (Goto et al. 2015).

### Biocathode

Biocathode offers biocompatible, cost-effective, and promising material for many applications such as heavy metal removal and waste treatment. The use of biocathodes eliminates the need for expensive construction material and potentially toxic chemicals as catholyte, and further the necessity for their recycling and safe disposal (Gude et al. 2013). Algae play a crucial role in nitrogen and phosphorus cycles in waters. The use of algae to produce oxygen is being considered for exploiting its feasibility as an oxygen supplier for cathodic reaction in MFCs. In order, the produced CO_2_ by anode through biomass oxidation would be transferred to the cathode as a carbon source for algae growth by the photosynthesis process. Cui et al. (2014) utilized *Chlorella vulgaris* as biocathode. The maximum PD, and CE at 2500 mg COD/L could be obtained 1926 ± 21.4 mW/m^2^ and 6.3 ± 0.2%, respectively. The use of biocathode would significantly reduce the cost of MFC and expand its applications (e.g., CO_2_ fixation, microalgae cultivation) in biomass-fueled MFC.

### Genetically engineered microorganisms

Molecular biology techniques helped to clarify the pathways for  electron transfer steps and also provide the possiblity to  engineer microorganisms to use  biomass as fuels for electricity generation. So far, various technical approaches including random approaches (i.e., directed evolution of redox enzymes, and silver/gold coating of cells), rational design (i.e., heterologous gene expression, engineering of metabolic processes, and engineering of bacterial pili), and de-novo design (i.e., bacterial surface display of redox proteins, yeast surface display of redox proteins, and hybrid MFC-enzyme based fuel cells) have been investigated for the genomic engineering of a novel or optimized biocatalysis in MFCs (Alfonta 2010; Zhao et al. 2020). Recently, Li et al. (2019) designed a bioengineered microbial consortium of *Klebsiella pneumonia*–*S*. *oneidensis* for efficiently harvesting of the electricity from corn stalk hydrolysate. The eliminating of the ethanol and acetate pathway via deleting *pta* (phosphotransacetylase gene) and *adhE* (alcohol dehydrogenase gene) genes could reinforce the lactate production in *K. pneumoniae*. Also, a biosynthesis and delivery system for transporting lactate was assembled in this strain through expressing *ldhD* (lactate dehydrogenase gene) and *lldP* (lactate transporter gene). Thus, the engieered *K. pneumoniae* could ferment the hydrolysate to lactate as fuel for electricity generation by *S. oneidensis*. Furthermore, to improve the EET efficiency of *S. oneidensis*, a heterogenous  flavins synthesis pathway from *Bacillus subtilis* was expressed in *S. oneidensis. *The genetically engineered microbial consortia showed high efficiency for electricity generation from biomass hydrolysate (Li et al. 2019). These findings demonstrate genetic engineered microorganisms would be promising to be adapted for biomass-fueled MFCs.

### Coproduction of electricity with other energy products

It is well known that biorefinery of biomass only can harvest a limited fraction of the energy in the biomass, leading to low energy efficiency and a large quantity of wastes. It is a highly innovative approach in MFCs that electricity can be coproduced with other energy products, which can substantially improve the overall energy efficiency for biomass conversion with MFCs. It is reported that MFCs can further recovery the energy from the waste of biomass biorefinery (Offei et al. 2019). For example, a novel biorefinery approach involved the coproduction of bioethanol and electricity production from tropical seaweeds has been developed (Offei et al. 2019). The seaweed biomass was first used for bioethanol fermentation and then the bioethanol production residues were employed for bioelectricity generation in MFC. This combination process achieved coproduction of bioethanol as high as 5.1 g/100 g dry biomass and 0.5 W/m^3^ power density, which also reduced waste to 24.4% from 69 to 79% for seaweed bioethanol production alone. More recently, the strategy of isolation and acclimation of a new exoelectrogenic yeast strain (*Cystobasidium slooffiae* JSUX1) could produce significant bioelectricity and biohydrogen production simultaneously with rapid xylose (secondary dominant sugar derived from biomass) metabolism in MFC, in which the produced electrons were harvested from H_2_ fermentation with xylose (Moradian et al. 2020). The coproduction of electricity and H_2_ is of great interest for application as these two energy products are obtained in a single operated MFC, which would significantly reduce the cost of reactor and operation.

## Conclusions and perspectives

Biomass is one of the most abundant renewable energy resources on the earth. Conversion of the biomass directly into the most conventional electric energy is considered a promising roadmap for the biomass energy industry. In recent years, it was found that MFCs could directly convert the biomass into electricity with high energy efficiency, which could bypass the Carnot limitation. Therefore, various biomass-fueled MFCs have been developed and were extensively studied. Raw biomass is hard to be metabolized by EAMs. Thus, a variety of pretreatment techniques employed to degrade the lignocellulosic biomass and release the carbohydrates products. The integration of the pretreatment steps not only improve CE, but also broaden the substrate spectrum for biomass-fueled MFCs. Since EAMs are the key player for energy conversion in MFCs, various types of EAMs including Gram-negative and Gram-positive bacteria, yeast, fungi, and mixed-culture from activated sludge were used as inoculum for biomass-fueled MFCs. Usually, unsuitable EAMs for biodegradation/biorefinery in MFCs cause a large quantity of non-oxidized substrates, which results in a low CE output in MFCs. Therefore, the selection of a suitable EAM and selection of preferred substrate are suggested for improved EC of biomass-fueled MFCs. Further, for complex biomass, constructing MFCs with diverse biocatalysis of EAMs in a single MFC can be suggested for enhancing CE in biomass-fueled MFCs. Also, operation conditions and optimization can significantly affect the performance of biomass-fueled MFCs. The current review focused on optimization parameters for MFCs performance improvement, while insights into the mechanism for biomass energy conversion and electron transportation are still very limited, which requires further exploration. Besides, detailed Omics and functional analysis, genome mining of the microbial community for biomass-fueled MFCs are expected, which holds a great promise to explore new EAMs species and new functional genes/clusters/proteins. Moreover, synthetic consortium or artificial superbug integrating the functions of biomass degradation, saccharification, and bioelectricity conversion are expected to be developed, which would result in a consolidated process and surely simplify the process for biomass-fueled MFCs. Systematic process optimizations with special consideration of the unique features of biomass fuels and practical applications are still required. Finally, new process design and scale-up research are urgently needed, which would be essential for practical applications.

## Data Availability

Not applicable.

## Abbreviations

BDMs: Biomass-degrading microorganisms
CA: Concentrated mineral acid
CC: Carbon cloth
CDBs: Cellulose-degrading bacteria
CE: Coulombic efficiency
CF: Carbon felt
CNTs: Carbon nanotubes
COD: Chemical oxygen demand
DA: Dilute mineral acid
DMMFC: Dairy manure microbial fuel cell
DOM: Dissolved organic matters
EAM: Electroactive microorganism
EET: Extracellular electron transfer
EIS: Electrochemical impedance spectroscopy
GO: Graphene oxide
HRT: Hydraulic retention time
HTL: Hydrothermal liquefaction
KWADE: Kitchen waste anaerobically digested effluent
MFC: Microbial fuel cell
OLR: Organic loading rate
PANI/CNC: Polyaniline/crystalline nanocellulose
PD: Power density
PEM: Proton exchange membrane
PMFC: Plant microbial fuel cell
PPy: Polypyrrole
rGO: Reduced graphene oxide
SMFC: Sediment microbial fuel cell
